# A rabbit osteomyelitis model for the longitudinal assessment of early post-operative implant infections

**DOI:** 10.1186/1749-799X-8-38

**Published:** 2013-11-04

**Authors:** Jim CE Odekerken, Jacobus JC Arts, Don AM Surtel, Geert HIM Walenkamp, Tim JM Welting

**Affiliations:** 1 Laboratory for Experimental Orthopaedics, Department of Orthopaedic Surgery, CAPHRI School for Public Health and Primary Care, Maastricht University Medical Centre, P.O. Box 5800, Maastricht 6202 AZ, the Netherlands

**Keywords:** Implant infection, Osteomyelitis, Animal model, Rabbit, *Staphylococcus aureus*

## Abstract

**Background:**

Implant infection is one of the most severe complications within the field of orthopaedic surgery, associated with an enormous burden for the healthcare system. During the last decades, attempts have been made to lower the incidence of implant-related infections. In the case of cemented prostheses, the use of antibiotic-containing bone cement can be effective. However, in the case of non-cemented prostheses, osteosynthesis and spinal surgery, local antibacterial prophylaxis is not a standard procedure. For the development of implant coatings with antibacterial properties, there is a need for a reliable animal model to evaluate the preventive capacity of such coatings during a specific period of time. Existing animal models generally present a limited follow-up, with a limited number of outcome parameters and relatively large animal numbers in multiple groups.

**Methods:**

To represent an early post-operative implant infection, we established an acute tibial intramedullary nail infection model in rabbits by contamination of the tibial nail with 3.8 × 10^5^ colony forming units of *Staphylococcus aureus*. Clinical, haematological and radiological parameters for infection were weekly assessed during a 6-week follow-up with *post*-*mortem* bacteriological and histological analyses.

**Results:**

*S. aureus* implant infection was confirmed by the above parameters. A saline control group did not develop osteomyelitis. By combining the clinical, haematological, radiological, bacteriological and histological data collected during the experimental follow-up, we were able to differentiate between the control and the infected condition and assess the severity of the infection at sequential timepoints in a parameter-dependent fashion.

**Conclusion:**

We herein present an acute early post-operative rabbit implant infection model which, in contrast to previously published models, combines improved in-time insight into the development of an implant osteomyelitis with a relatively low amount of animals.

## Background

The implantation of orthopaedic prostheses/trauma implants is an invasive surgical procedure with an increased risk of post-operative infections compared to non-implant-related orthopaedic interventions. Since the lifespan and quality of orthopaedic implants are gradually improving and more biomaterials are implanted every year, the prevalence of post-operative infections is expected to increase [1-3].

Most orthopaedic implant infections and osteomyelitises are of staphylococcal origin [3-5]. The most frequent direct post-operative (acute) deep implant infections are the result of trauma, while infection after a total joint replacement is less frequent [6,7]. Antibiotic treatment of these infections often fails due to biofilm formation by the infecting pathogen on the implant surface [6,7].

Most orthopaedic implants are based on titanium or cobalt-chrome alloys, and their overall biocompatibility is good. However, these materials do not possess antimicrobial properties. Applying antimicrobial functionality to an implant, e.g. using coatings to prevent biofilm formation, could provide an effective way to minimize the risk of bacterial colonization. For evaluating the efficacy of novel antimicrobial implant coatings, *in vivo* testing is essential to establish a reliable coating, suitable for orthopaedic implants.

One of the most well-known *in vivo* models is the model by Norden [8], in which rabbits receive an intramedullary contamination of *Staphylococcus aureus* after the administration of a sclerosing agent (sodium morrhuate). To mimic the presence of an orthopaedic implant, the rabbit model by Andriole et al. [9,10] introduced an intramedullary pin in a contaminated tibial intramedullary cavity. Over the years, these models have been modified for the clinical evaluation of implant materials and coatings [11-16] or for the evaluation of antibiotic treatment efficacy, like antibiotic-containing bone cements [14,17-19]. Despite the many published rabbit infection models, these studies seldomly report on time course measurements of infection parameters in the same animal in follow-up studies. Determining infection parameters in individual animals over time is expected to provide important information about the development of osteomyelitis and the in-time bone integrity surrounding the implant, as well as bone apposition dynamics on the implant. Furthermore, such an experimental setup would also require less animals without making any concessions on the experimental significance (according to the 3R guidelines proposed by Russell and Burch [20].

To address the current lack of a model providing data on the above parameters in individual animals during the course of osteomyelitic development, we aimed to establish an improved rabbit implant infection model, based on several previously published models resembling clinical orthopaedic implant infections [8-10,12,15]. By combining haematological parameters (leucocyte differentiation, erythrocyte sedimentation rate (ESR) and C-reactive protein (CRP)) with clinical parameters (body weight and body temperature) and bone integrity parameters (determined by X-ray, micro-computed tomography (CT) and histology) during a 6-week follow-up in rabbit osteomyelitic development, a broad-spectrum insight into the development of the infection status in individual subjects was acquired, using a minimal number of animals.

## Methods

### Inoculum preparation

The *S. aureus* strain UAMS-1 (a clinical methicillin-sensitive isolate, obtained from the American Type Culture Collection (ATCC 49230, ATCC, Manassas, VA, USA)) [5,21] was used in the experiments. Bacteria were cultured in tryptic soy broth (Bacto, Becton Dickinson, Pont-de-Claix, France). A bacterial inoculum was created from a fresh overnight culture which was subsequently diluted with sterile saline to a concentration within the range of 1 × 10^6^–1 × 10^7^ colony forming units (CFU)/ml, based on OD600 (Amersham Biosciences, GE Healthcare, Piscataway, NJ, USA) measurements. The range of the inoculum was based on our previous *in vivo* dose finding experiments in New Zealand White rabbits (data not shown). A contamination with 100 μl of this inoculum generally led to distinctive clinical and bone morphological changes related to osteomyelitis, with limited cases of spontaneous remission. The bacterial count of every inoculum was verified by quantitative culture on tellurite glycine agar (Difco, Becton Dickinson, Pont-de-Claix, France) before and after surgery. The inoculum size used in this study was 3.8 × 10^5^ CFU per contamination.

### Surgery and animal welfare and health

Twenty-two specific pathogen free (SPF) female New Zealand White (NZW) rabbits (Charles River, L'Arbresle, France), with a weight of 3.5–4 kg (approximately 6 months of age), were used in this study. After arrival, the animals were allowed to acclimatize for 2 weeks before surgery was performed.

Anaesthesia was initiated using ketamine (35 mg/kg intramuscularly (i.m.); Nimatek, Eurovet Animal Health, Bladel, the Netherlands) and xylazine (5 mg/kg i.m.; Xylalin, Ceva Santé Animale, Libourne, France) while maintained by fentanyl (2 μg/kg/h intravenously (i.v.); Fentanyl, Hameln Pharmaceuticals, Hameln, Germany) and midazolam (1 mg/kg/h i.v.; Midazolam, Actavis, Zug, Switzerland) and if necessary supported by isoflurane (1 %, Isoflo, Abbott Laboratories, Abbott Park, IL, USA). The right hind leg of the animals was clipped (not shaved) and disinfected with 2 % iodine solution (Eurovet Animal Health, Bladel, the Netherlands).

**Figure 1:**
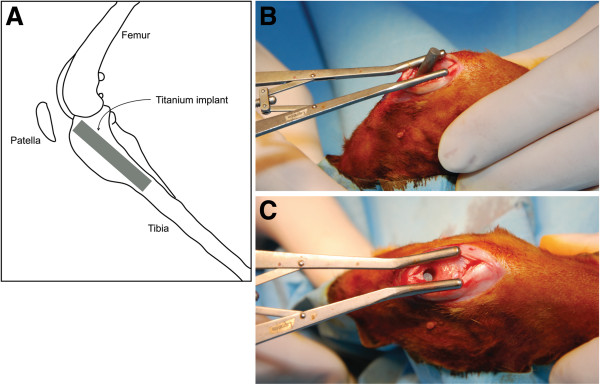
**Implant localization and implantation. (A)** Localization of the titanium implant in the proximal tibia. **(B)** Transpatellar insertion of the implant. **(C)** Implant positioning after implantation, before sealing with bone wax.

The animals were randomly assigned to two separate surgical groups (based on a power calculation according to Sachs [22]): a contamination group (*n* = 11) and a sterile saline control group (*n* = 11). By sequential reaming, a 4-mm-wide defect was drilled by hand into the tibial plateau to open the tibial medullary canal. After reaming, the tibial medullary cavity was flushed with sterile saline to remove bone fragments and haematoma. Each animal received a 20-mm-long, 4-mm-wide grit-blasted titanium nail (TiAl6V4, DePuy, Warsaw, IN, USA) in the proximal part of the tibia by transpatellar incision (Figure 1A).

Immediately before insertion of the implant, the contamination group received an intramedullary contamination of 3.8 × 10^5^ CFU of *S*. *aureus* in 100 μl saline. In the control group, 100 μl saline was administered. The implant was press-fit into the tibial defect (Figure 1B) and positioned just below the tibial articular cartilage surface (Figure 1C). After insertion, the defect was sealed with bone wax (Syneture, Covidien, Mansfield, MA, USA) and the surrounding tissue was flushed with sterile saline as an extra prophylaxis for soft tissue infection. The wound was closed in layers with resorbable sutures (Syneture, Covidien, Mansfield, MA, USA). Furthermore, aluminum spray (Eurovet Animal Health, Bladel, the Netherlands) was applied to protect the wound.

For the first 2 days after surgery, the animals were treated with subcutaneous injection of buprenorphine (twice a day, 0.05 mg/kg body weight, Temgesic, Reckitt Benckiser, Slough, UK). Pain treatment, with buprenorphine, was continued if pain persisted after these 2 days.

The animals were housed in groups to promote movement of the operated leg. Housing groups were based on the random assignment of the animals to a specific operating day, resulting in mixed housing groups containing both infected and uninfected animals. The animals were monitored during the 6-week follow-up for the use of their hind legs, the appearance of the wound and general signs of infection (redness, swelling and fever). Food and water were available *ad libitum*. The daily diet was supplemented with Critical Care (Oxbow Animal Health, Murdock, NE, USA).

Body weight and temperature were measured pre-operatively on the day of surgery and every week thereafter until the end of the experiment. Blood was collected by venipuncture from the jugular vein of the rabbit from which approximately 2 ml of blood was drawn to determine leucocyte differentiation (Euregio Laboratory, Maastricht, the Netherlands), ESR (Kabe Labortechnik, Nümbrecht-Elsenroth, Germany) and CRP (E-15CRP, Immunology Consultants Laboratory, Portland, OR, USA).

Tibial fracture, soft tissue infection with a large abscess or fistula and sepsis were defined as humane endpoints which would directly lead to termination of the animal. In case of 20 % weight loss, a veterinarian was consulted and the animal treated accordingly (either euthanization or additional individual feeding with Critical Care). According to the experimental procedure, all remaining animals were sacrificed 6 weeks after surgery by a pentobarbital (Euthanimal, Alfasan Diergeneesmiddelen, Woerden, the Netherlands) overdose.

This study was approved by the Maastricht University Animal Ethical Committee (DEC-UM, Protocol 2010–089, Maastricht, the Netherlands). Dutch law guidelines for animal experiments were strictly followed while designing and conducting this study.

### Imaging

Antero-posterior and medio-lateral X-rays of the tibia were made under tiletamine-zolazepam sedation (15 mg/kg i.m., Zoletil 100, Virbac Laboratories, Carros, France) at 85 kV and 20 mAs (Polymobil, Siemens, Erlangen, Germany) on Kodak PQ-phosphor screens (Carestream Healthcare, Rochester, NY, USA) with a phosphor screen-to-source distance of approximately 70 cm. Data were digitized using a CR-900 plate reader (Carestream Healthcare, Rochester, NY, USA). Images were assessed with the Philips iSite (version 3.5) software package (Royal Philips Electronics, Eindhoven, the Netherlands). Modified X-ray scoring systems (based on the classification by Calhoun and Mader [17]) were used for describing the specific changes around the infected intramedullary implant in a rabbit (Tables 1 and 2). All individual X-ray radiographs were scored (according to Table 1) by three independent, blinded observers. *Ex vivo* micro-CT imaging of the implant and the surrounding area was performed on the excised tibiae after the 6-week follow-up. The micro-CT images were acquired on an X-rad 225 (Precision X-ray, North Branford, CT, USA), with a field of view of 10 cm in diameter, a source-to-axis distance of 30 cm and a source-to-detector distance of 62 cm. Images were made at 80 kVp, with an isotropic spacing of 102 μm and 2.14 mm AI added filtration. Data were assessed with the GE MicroView software package (version 2.1.2, GE Healthcare, Pewaukee, WI, USA). Individual micro-CT images were scored by three independent, blinded observers (according to Table 2).

**Table 1:** **Osteomyelitis scoring system**—**X**-**ray**

**Osteomyelitis grade**	**Morphological changes**
0	No radiologic abnormalities
1	Mild periosteal reaction
	Mild osteolysis directly around the implant
2	Periosteal reaction
	Evident osteolysis around the implant
3	Periosteal reaction with subperiostal calcification
	More extensive metaphyseal osteolysis
4	Cortical thickening
	Osteolysis extending into diaphysis

**Table 2:** Osteomyelitis scoring system—micro-CT

**Osteomyelitis grade**	**Morphological changes**
0	No radiologic abnormalities
1	Mild periosteal reaction
	Mild cortical thickening
2	Evident periosteal reaction
	Evident cortical thickening
	Mild osteolysis
3	Extensive cortical thickening
	Focal loss of cortical wall
	Evident osteolysis
4	Extensive cortical thickening
	Loss of cortical morphology
	Loss of spongeous morphology
	Extensive osteolysis

### Calcium-binding fluorophores

Three different calcium-binding fluorophores were administered, by subcutaneous injection, to follow bone apposition and mineralization over time. At week 2 of follow-up, 25 mg/kg calcein green (Fluka, Sigma-Aldrich, Seelze, Germany) was injected, 30 mg/kg xylenol orange (Fluka, Sigma Aldrich, Seelze, Germany) was injected at week 4 and 25 mg/kg calcein blue (Fluka, Sigma Aldrich, Seelze, Germany) was injected on day 41 (the day before sacrifice), resulting in green, orange and blue zones, indicating active bone formation at that particular timepoint.

### *Post-mortem* bacterial culture

After sacrifice, the tibiae were dissected aseptically. Swabs were taken from the knee joint cavity and tibial plateau. To assess soft tissue infection, swabs were evaluated for the presence of *S*. *aureus* on tellurite glycine agar plates. A 5-mm piece of the distal part of the *tuberositas tibiae* was excised from the tibia with a surgical drill (SM 12, Nouvag, Goldach, Switzerland). After weight measurement, it was homogenized (Ultra-Turrax T25, Ika, Staufen, Germany) and cultured on tellurite glycine agar plates. After 24 h, culture dishes were quantified for specific bacterial growth.

### Histology

After sampling for bone culture, tibiae were fixated in 4 % formaldehyde/PBS and subsequently embedded in polymethyl methacrylate (PMMA; Technovit 9100, Heraeus Kulzer, Hanau, Germany). Fifty-micrometer-thick sections were prepared using a saw microtome (Leica SP1600, Wetzlar, Germany), and each section was stained according to Masson-Goldner (Carl Roth, Karlsruhe, Germany) or Gram (without safranin O counterstain). Sections were analyzed and digitized by light microscopy (Axioscope A1, Axiovision LE release 4.8.2, Carl Zeiss, Oberkochen, Germany). The localization of calcium-binding fluorophores in the bony tissue was visualized by fluorescence microscopy (Leica DMRB, Leica IM50 version 1.2 release 19, Leica, Wetzlar, Germany) on unstained PMMA sections. Acquired images were merged using Photoshop CS3 (Adobe Systems, San Jose, CA, USA) to generate overview images. Histological sections were scored by three blinded, independent observers (according to Table 3).

**Table 3:** Osteomyelitis scoring system—histology

**Morphological abnormality**	**Histological staining**	**Score (per abnormality)**
Cortical thickening	Masson-Goldner	0: absent
Presence of micro-abscesses		1: mild to moderate
Enlarged Haversian canals		2: moderate to severe
Periosteal elevation	Fluorescent calcium indicators	
Gram stain	Modified Gram stain	0: negative
		2: positive

Total histological score and osteomyelitis grade: 0–3, no osteomyelitis; >3–5, mild; >5–7, moderate; >7–10, severe.

### Statistical analysis

SPSS 19 (IBM, Armonk, NY, USA) was used for the statistical analyses. Data were checked for normality using the Shapiro-Wilk test. Differences between groups were determined by a Mann–Whitney *U* test for non-parametric one-tailed significance. The significance level was determined at *p* < 0.05. Graphical representation of the data was performed in GraphPad Prism 5 (GraphPad, San Diego, CA, USA).

## Results

### Surgery and follow-up

**Figure 2:**
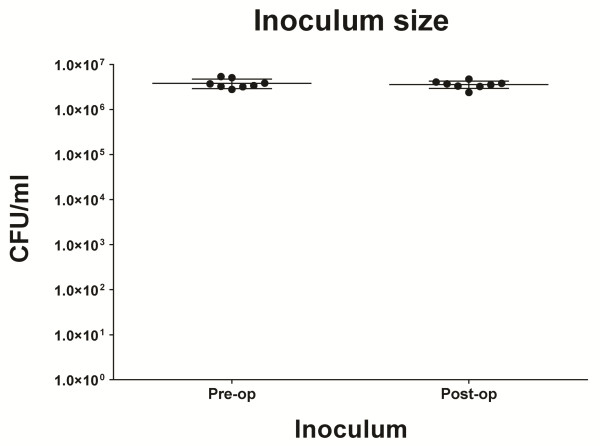
Inoculum size verification before and after surgery.

In total, 22 rabbits received an implant by a transpatellar incision, of which 11 received an intramedullary inoculation with 3.8 × 10^5^ CFU of *S*. *aureus*. The inoculum size was checked before and after surgery to ensure a constant intraoperative bacterial load (Figure 2). Due to respiratory depression, three rabbits did not recuperate from anaesthesia during the follow-up (two animals were part of the control group and one of the contaminated implant group). Another three animals (all from the contaminated implant group) had to be sacrificed during follow-up due to humane endpoint complications. Blood cultures were taken after sacrifice to exclude sepsis. All blood cultures were negative for *S*. *aureus* and *Staphylococcus epidermidis*. In total, these six animals, including the corresponding data, were considered as lost to follow-up, resulting in a control group of nine animals and a contaminated implant group of seven animals.

### Physical examination and haematological analysis

**Figure 3:**
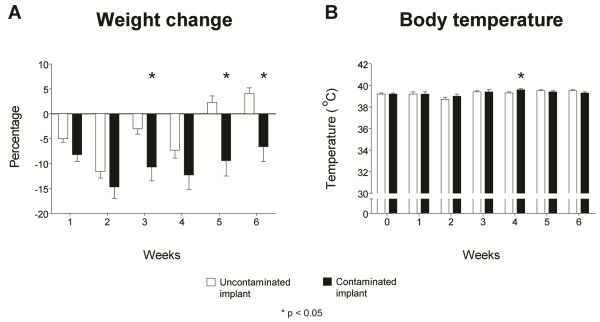
**Physiological parameters. (A)** Weight change during follow-up. **(B)** Body temperature during follow-up. *White bars* represent the control population while *black bars* represent the contaminated implant group. *Error bars* represent the standard error of the mean. *Asterisk* indicates *p* < 0.05.

After recuperation, the control group returned to full weight bearing of the operated leg in the first week after surgery, while most animals in the contaminated implant group did not recover to full weight bearing within the 6-week follow-up period. Weight loss was noted in the first weeks after surgery in both groups. The contaminated implant group had significantly more weight loss compared to the uncontaminated control implant group at the third, fifth and sixth post-operative week (*p* = 0.071, 0.176, 0.028, 0.071, 0.006 and 0.006, respectively) (Figure 3A).

There was no incidence of fever in both groups. The temperature range of the control group was 37.7°C–39.9°C, while that of the contaminated implant group was 37.9°C–39.8°C. The body temperature in the contaminated implant group was significantly higher compared to that in the uncontaminated implant group at the fourth post-operative week only (*p* = 0.046) (Figure 3B).

**Figure 4:**
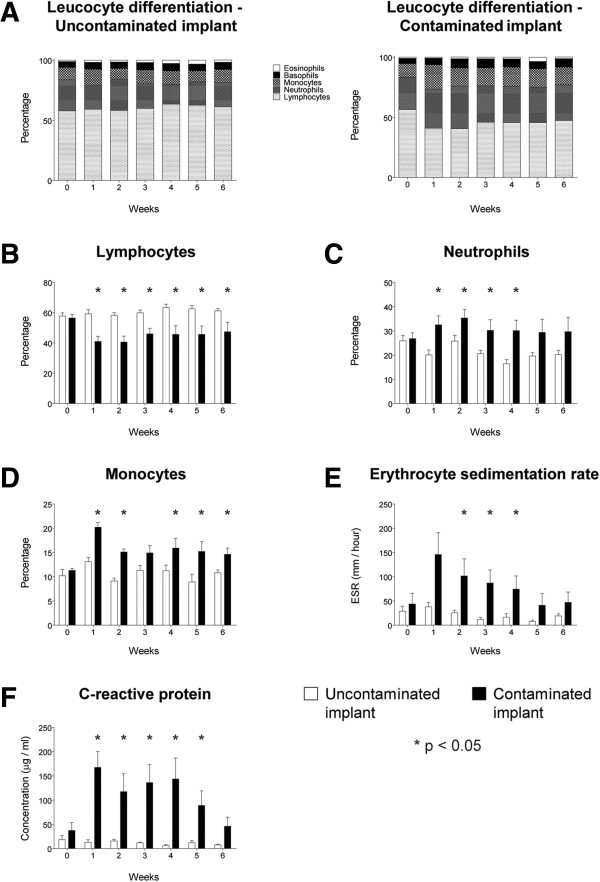
**Haematological parameters. (A)** Summary of the leucocyte differentiation. **(B)** Percentage of lymphocytes. **(C)** Percentage of neutrophils. **(D)** Percentage of monocytes. **(E)** Erythrocyte sedimentation rate. **(F)** The C-reactive protein levels. *White bars* represent the control population while *black bars* represent the contaminated implant group. *Error bars* represent the standard error of the mean. *Asterisk* indicates *p* < 0.05.

The leucocyte differentiation in the control group remained unchanged throughout follow-up, while the contaminated implant group presented with a clear shift in differentiation after surgery, compared to the pre-operative and control group levels. During follow-up, the contaminated implant group showed a relative decrease of the percentage of lymphocytes in favour of neutrophil and monocyte fractions (Figure 4A), resulting in a significantly lower lymphocyte percentage in the contaminated implant group compared to the controls in the entire follow-up (*p* = 0.001, 0.001, 0.001, 0.001, 0.003 and 0.021, respectively) (Figure 4B). Neutrophilic granulocyte fractions were significantly higher in the first four post-operative weeks only (*p* = 0.010, 0.047, 0.016, 0.001, 0.055 and 0.105, respectively) (Figure 4C). The percentage of monocytes in the contaminated implant group was significantly higher at nearly all post-operative weeks except for the third post-operative week (*p* = 0.001, 0.0001, 0.055, 0.028, 0.016 and 0.006, respectively) (Figure 4D).

The ESR was significantly higher in the contaminated implant group at the second, third and fourth weeks (*p* = 0.076, 0.012, 0.006, 0.036, 0.051 and 0.126, respectively) as compared to the uncontaminated control group (Figure 4E). Plasma CRP levels were specifically elevated in the contaminated implant group, for up to 5 weeks after surgery (*p* = 0.0001, 0.021, 0.0001, 0.001, 0.012 and 0.057, respectively) (Figure 4F).

### Radiology

**Figure 5:**
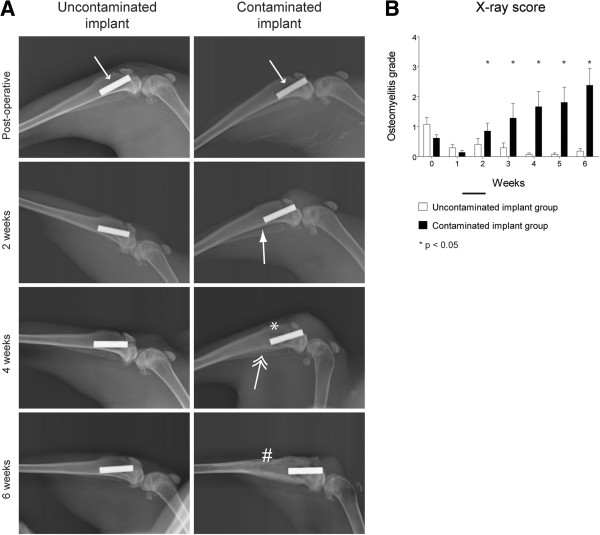
**Quantitative radiological *in vivo *imaging. (A)** X-ray images taken during follow-up. The *small arrowhead* at the post-operative images indicates the possible inter-operative damage to the peri-implant tissue. The *solid arrow* at 2 weeks indicates the presence of a periosteal reaction. The *asterisk* at 4 weeks indicates metaphyseal osteolysis, while the *double arrowhead* points at calcification of the periosteal reaction noted at 2 weeks. The *hash sign* at 6 weeks indicates diaphyseal osteolysis. **(B)** Quantification of the X-ray images. *White bars* represent the control population while *black bars* represent the contaminated implant group. *Error bars* represent the standard error of the mean. *Asterisk* indicates *p* < 0.05.

Several clinical radiological parameters for infection, like bone morphological changes, periosteal elevation and periosteal thickening together with meta- and diaphyseal osteolysis were observed on X-ray radiographs, enabling the use of our modified scoring system for osteomyelitis (Tables 1, 2, and 3). After 6 weeks of follow-up, the control group showed no signs of osteomyelitis or abnormal morphology of the bone tissue with correct implant placement. Still, radiologically visible artefacts from the surgical procedure may suggest the presence of osteolysis around the implant in the direct post-operative images (Figure 5A, small arrowhead). The contaminated implant group however showed the first signs of osteomyelitis with periosteal reactivity at 2 weeks after surgery (Figure 5A, solid arrowhead). Osteolysis starting at the metaphyseal level and calcification of the initial periosteal reaction were observed around the fourth week (Figure 5A, asterisk and double arrowhead, respectively). Diaphyseal osteolysis, with resorption of the cortex and involucrum formation, was generally observed at the sixth week (Figure 5A, hash sign). Scoring of the individual timed radiographs, using the modified osteomyelitis scoring system (Table 1), resulted in a significantly higher score for animals with a contaminated implant from the second post-operative week onwards (*p* = 0.204, 0.046, 0.036, 0.003, 0.0001 and 0.001, respectively) (Figure 5B). This indicates that infected and uninfected implants can be distinguished in this model from 2 weeks after surgery.

**Figure 6:**
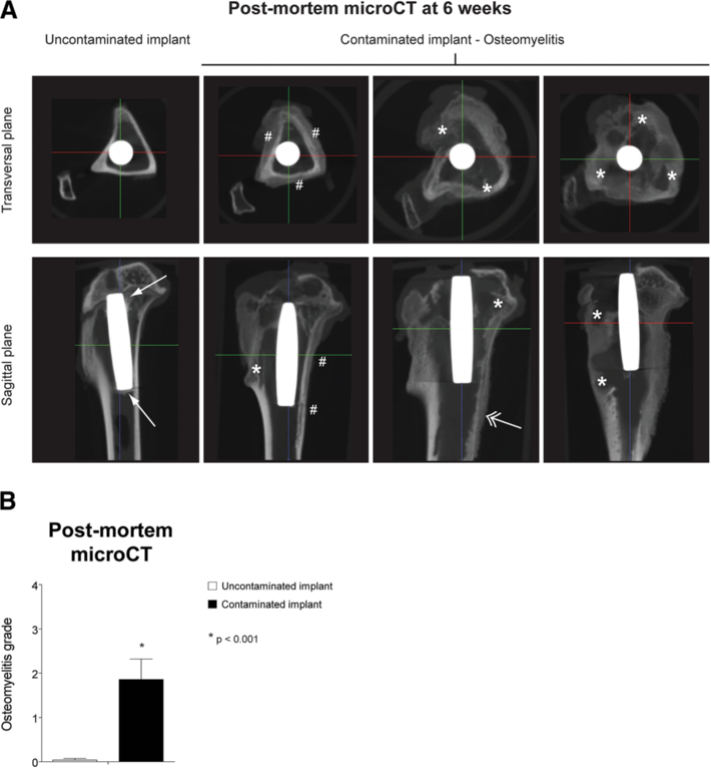
**Quantitative radiological *ex vivo *imaging. (A)** Representative *post-mortem* micro-CT images taken after the 6-week follow-up. The *hash sign* indicates calcification of the periosteal reaction, the *asterisk* indicates osteolysis, the *solid arrow* points at bone apposition at the implant surface and the *double-headed arrow* indicates the presence of an involucrum. **(B)** Quantification of the micro-CT images allows differentiation between the control group (*white bar*) and the implant infection group (*black bar*). *Error bars* represent the standard error of the mean. *Asterisk* indicates *p* < 0.001.

**Figure 7:**
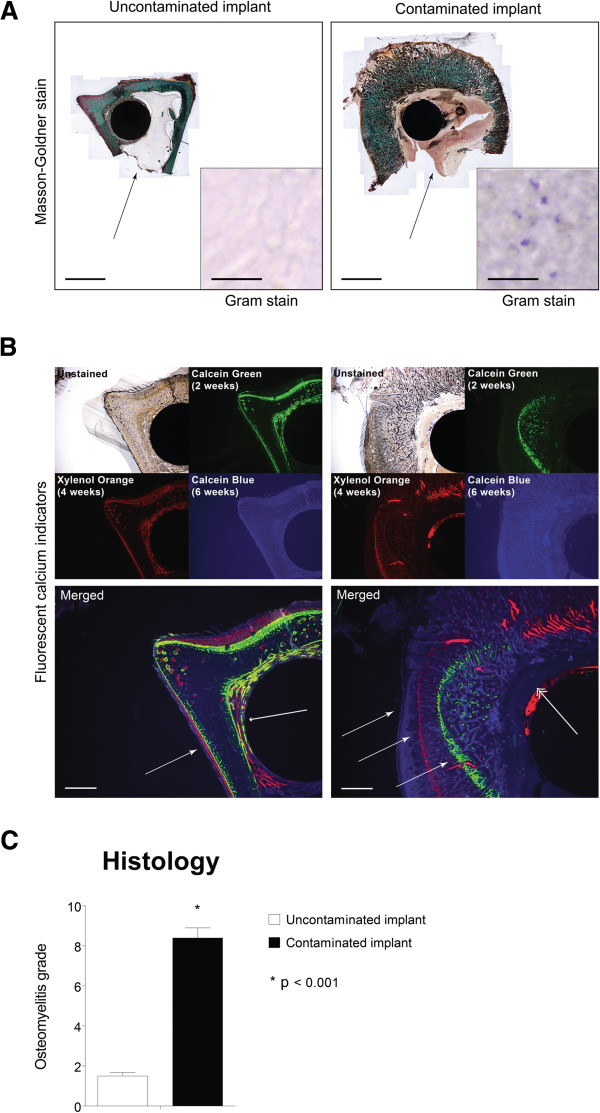
**Quantitative histology. (A)** Histological sections of the distal part of the implant region. Sections show distinctive morphological changes in the contaminated implant group, with bone apposition on the implant surface in the uncontaminated implant group and cortical thickening in the contaminated implant group. *Black arrow* indicates an intramedullary abscess (*bars* represent 4 mm). Gram staining (*insert*) shows Gram-positive cells in the contaminated implant group while the uncontaminated group remains negative (*bars* represent 20 μm). **(B)** Fluorescent calcium indicators represent calcium deposition at the time of injection, indicating bone development (*large arrowhead*) and implant ingrowth (*small arrowhead*) in the uncontaminated implant group. In the contaminated implant group, these indicators point at calcification of the periosteum due the periosteal elevation (*three identical white arrows*) caused by the presence of bacteria. The *double arrowhead* indicates osteolysis in the peri-implant tissue (*bars* represent 1 mm). **(C)** Quantification of the histological symptoms of implant infection shows a distinct, significant difference between the control group (*white bar*) and the infected implant group (*black bar*). *Error bars* represent the standard error of the mean. *Asterisk* indicates *p* < 0.001.

*Post-mortem* micro-CT imaging at 6 weeks was carried out to acquire in-depth overview of the implant/infection area in an axial direction, focussing on bone remodelling around the implant. The control group showed clearly mineralized cortices with sharp boundaries, correct implant positioning and bone apposition on the implant surfaces. No signs of osteolysis were observed. (Figure 6A, far left panels). All contaminated implants on the other hand showed distinctive infection characteristics visible on micro-CT (Figure 6A, right panels). Remodelling of the tibial cortex, metaphyseal and diaphyseal osteolysis, resorption of the tibial cortex, deformity of the tibial plateau and limited to no bone apposition on the implant were observed. Furthermore, while the sagittal planes allowed comparison with the X-ray radiographs (Figure 5), the transversal planes allowed comparison between the histological sections and provided more information on the axial direction with regard to histomorphological changes in the peri-implant tissue (Figure 7). Blinded scoring of the micro-CT images (Table 2) allowed quantification of the osteomyelitic status of the rabbit tibia after the 6-week follow-up period. This resulted in a significant differentiation between the control group and the infected implant group (*p* = 0.0001) (Figure 6B).

### Bacterial culture

**Figure 8:**
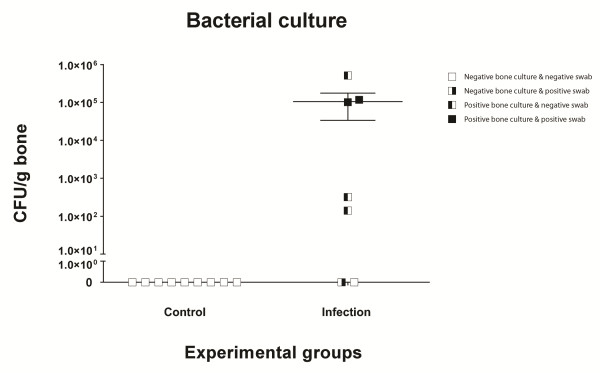
***Post****-****mortem *bacterial culture.** Swabs and bone samples were obtained from each animal and cultured on tellurite glycine agar. The graph indicates culture negativity in all control group samples, while six out of seven animals of the contaminated implant group cultured positive for *S*. *aureus* infection.

*Post-mortem* tissue swabs and bone homogenates were selectively cultured on tellurite glycine agar to determine the presence of *S*. *aureus* and other bacterial species. All swabs and bone homogenates taken from control group animals were negative for bacterial growth, while samples from six out of seven animals of the contaminated implant group were positive for *S*. *aureus* growth (Figure 8). No growth of *S*. *epidermidis* was detected in these samples.

### Histology

Masson-Goldner-stained sections revealed normal tibial cortex morphology, bone apposition around the titanium implant and no indication of bacterial presence in PMMA sections of all control tibiae (Figure 7A, left panel). This was in sharp contrast to every contaminated tibia, where Masson-Goldner-stained sections show the destructive effect of the infection by cortical thickening, absence of implant ingrowth, abscesses in the bone marrow cavity and enlarged Haversian canals (Figure 7A, right panel). A modified Gram staining was performed to address the presence of bacterial cells (live or dead). Confirming an infected state of the tibiae, Gram-positive cocci were found in all contaminated tibiae, whereas these were not microscopically detectable in control tibiae (Figure 7A, inserts). Three different fluorescent calcium indicators (Figure 7B, upper panels) allowed tracking of active bone apposition and remodelling on specified timepoints during the follow-up period. Fluorescent signals from the three different fluorophores were detected in sections of control tibiae, where the cortex showed sharp fluorescent linings with overlapping regions, as well as clear signals around the implant surface (Figure 7B, lower left panels). This indicates local bone apposition and remodelling in an organized and confined manner. In the contaminated tibiae, all fluorescent signals were also found, however showing highly disorganized patterning, representing mineralizing periosteal elevation and local remodelling due to osteolytic processes (Figure 7B, lower right panels). The modified scoring system (Table 3) allowed quantitative discrimination of the osteomyelitic status, between uncontaminated and contaminated tibiae. Scoring the histological sections according to this system resulted in two distinct groups that were significantly different (*p* = 0.001) and fully consistent with the contaminated and uncontaminated groups (Figure 7C).

## Discussion

Several animal models exist which generally focus on a select number of parameters for monitoring the activity of osteomyelitis. These models mainly focus on haematological parameters for comparing contaminated and control groups and others on radiological parameters in combination with histology [11,17,23] or bacterial culture [16,18,24-27]. The use of multiple experimental animal groups, sacrificed at pre-defined timepoints, is a frequently used method to specifically gain insight into infection development [28,29]. Yet, this approach requires a large number of animals. Our approach is partially based on previously published models [10,12,15], but we combined many of the most relevant infection parameters in one model and measured most of these parameters repeatedly throughout the 6-week follow-up in every animal. Besides lowering the number of required animals, this contributes to the dynamic analysis of the development of an implant infection in individual subjects over time. This broad collection of infection-related outcome parameters allows a selection to be made to establish tailored models in the future to evaluate novel antimicrobial coatings, to study implant fixation and bone apposition on the implant surface or to study the development of osteomyelitis or an implant infection. Still, some parameters were not included in our model, like the use of bioluminescent bacteria to induce the implant infection or the assessment of biofilm formation on the implant surface. These remain an option for dedicated models. Also, the use of an antibiotic-resistant strain, like MRSA, could further broaden the applicability of this acute model. However, as antibiotic-sensitive strains are most commonly found to cause orthopaedic implant infections in the clinic [3,30,31], at this stage we deliberately chose to establish this model with an antibiotic-sensitive strain. Debridement is a commonly used approach in the clinic to treat existing osteomyelitises. When this model will be used for the evaluation of novel therapeutic antimicrobial approaches like bioactive glass, antibiotic-containing bone fillers and cements, or resorbable microparticles [32,33], debridement should be included in this infection model as well. The herein described repeated use of anaesthetics in our model (seven episodes in total, for each rabbit) is not yet optimal since there is a risk of having an animal not recuperating from the anaesthesia. This was also recognized by Lankinen and colleagues [34]. Although kept to a minimum, this severe side effect could not be totally prevented in our present study. We lost three animals due to handling issues and respiratory depression related to the anaesthesia. These issues were considered as learning curve-related problems and might be prevented by the use of another combination of anaesthetics. A combination of ketamine-medetomidine-isoflurane should be considered for future experiments as a possible alternative with possibly a lower incidence of respiratory depression. Also, the loss to follow-up due to reaching humane endpoints (in our experiments, this only occurred in three animals of the contaminated implant group) is inevitable [12,25]. Still, daily checkup during follow-up and general supplementation of the daily diet with ‘Critical Care’ (extra fibres and vitamins) positively contributed to the overall animal health throughout the study. Furthermore, many studies based their experiments on individually caged animals [11-13,15,19]. We chose to house the animals in mixed groups. We regard this as an important aspect of this model because mixed group housing of the experimental groups stimulates the physical activity of the animals within a housing group and therefore encourages the use of the operated leg, thereby optimally maintaining the function of the operated limb.

The uncontaminated and contaminated groups could be distinguished relatively early by several systemic haematological parameters like ESR, CRP and leucocyte differentiation. As all haematological data consistently separated both groups early in the infection process, measuring a selected parameter may be sufficient for future animal studies in rabbits. For this purpose, we suggest using an ELISA-based measurement of plasma CRP levels as the most sensitive and optimal parameter to determine the presence of a developing implant infection. ESR and leucocyte differentiation can also be used in specific cases but may be more prone to experimental misinterpretation due to the used capillary approach or is relatively expensive, respectively.

In the development of our model, some limitations were encountered in the imaging of each animal over time. As an *in vivo* micro-CT was not at our disposal, we were not able to visualize bone development in all three dimensions over time *in vivo*. Instead, we applied weekly medio-lateral X-ray radiographs to visualize the tibiae in the sagittal plane over time and added *post-mortem* micro-CT to analyze the tibiae in three dimensions at the 6-week timepoint only. By combining the overtime X-ray imaging with the single timepoint micro-CT as well as histology and fluorescent imaging of the calcium-binding fluorophores, the overall bone development during follow-up could be visualized in great detail. Although true three-dimensional information on bone development over time is lacking, the herein described approach provides adequate information on bone activity for future interpretation of the efficacy of antimicrobial implant coatings.

Due to the complexity of the model, the need for clearly defined scoring systems for radiology and histology to allow proper discrimination of the infection status of an implant is evident. Several scoring systems have been described in the past, like the well-known X-ray scoring system of Calhoun and Mader [17] or the histological scoring system used by Petty and colleagues [35], modified by Vogely et al. [15]. Due to the high number of parameters in our study, these scoring systems could not be implemented in our model without necessary modifications. This resulted in three independent modified scoring systems for the assessment of X-ray radiographs, micro-CT and histology (Tables 1, 2 and 3). When developing these scoring systems, we used the original scoring systems [17,35] but incorporated additional results from X-ray radiographs, micro-CT and histology in our modified scoring systems. The X-ray radiograph and micro-CT scores are based on infection-induced morphological changes of the tibia, and the histological scoring system is a cumulative score based on the presence of multiple histomorphological infection parameters. Using the scoring systems on the herein described experimental groups, we were able to faithfully discriminate infected from uninfected tibiae. As detailed parameters were included in the scoring systems, we expect that these will be able to also detect more subtle changes due to, e.g. persisting low-grade infections.

Bacterial culturing of homogenized bone fragments and swabs showed specific growth of *S. aureus* in six out of the seven contaminated implants. The remaining implant was inconclusive for bacterial growth. This could be the result of clearing of the infection either by the host immune system or by stress acting on the bacterial cells during homogenization. This animal however also showed less severe radiological and histological symptoms of infection, while the damage to the peri-implant tissue and the haematological data in this contaminated animal indisputably pointed to an infection. As it seems that even within the same species identically administered bacterial contaminations do not develop equal infections [8,15,24], it stresses the use of multiple independent infection parameters to analyze the development of an osteomyelitic infection, to avoid large experimental groups and false negative scores based solely on negative bacterial cultures.

Although histological staining of PMMA sections clearly showed whether or not an infection was present in the tested tibiae ([12,15] and this study), analysis of this *post-mortem* material does not provide insight into infection-related bone remodelling over time. To overcome this hurdle, we additionally incorporated three different commonly used calcium-binding fluorophores in this animal model [36]. This allows assessment of bone remodelling/calcification caused by the initial infection and detection of subtle local changes that might be missed by other techniques. In our study both calcein green and xylenol orange were injected at earlier timepoints, whereas calcein blue was injected only 24 h before sacrifice. The short time frame before sacrifice could explain why the fluorescent signal for calcein blue was more diffuse as for the other two fluorophores (due to slow systemic clearance). This suggests that a calcium-binding fluorophore should be injected several days before sacrifice to obtain sharp fluorescent signals.

## Conclusion

The present study has shown that we established a pre-operative orthopaedic implant contamination model resulting in early post-operative infection in rabbits with a comprehensive multi-analytical 6-week follow-up. The modified scoring systems of several imaging techniques and histology allow a clear classification of the infection grade of the peri-implant tissue. The longitudinal assessment of the animals' infection status reduces the required number of test animals, without making concessions on the outcome data and statistics. In addition to the gained information on the longitudinal development of an acute implant infection, the above-mentioned suggestions (anaesthesia and nutritional supplementation) can improve future experimental survival. Our experimental and analytical setup may be used for the thorough longitudinal assessment of novel prophylactic antimicrobial coatings and antimicrobial treatments in a relatively small population of individual animals. Furthermore, our study suggests that a combination of weekly weight measurements, CRP and X-rays combined with biweekly injections of calcium-binding fluorophores and *post-mortem* bacterial bone culture provide the most optimal insight into infection development and status.

## Competing interests

This study was funded by the Dutch BioMedical Materials (BMM) program co-funded by the Dutch Ministry of Economic Affairs. This study is part of the BMM NANTICO Research project. The individual authors declare that they have no competing interests.

## Authors’ contributions

JO, GW and TW participated in the study design, data acquisition and analysis, and manuscript drafting and approved the manuscript. JA participated in the study design and manuscript drafting and approved the manuscript. DS participated in the data acquisition and analysis and approved the manuscript. All authors read and approved the final manuscript.
